# SEOM-GEMCAD-TTD Clinical Guideline for the diagnosis and treatment of esophageal cancer (2021)

**DOI:** 10.1007/s12094-022-02801-2

**Published:** 2022-03-26

**Authors:** Ana Fernández-Montes, Julia Alcaide, María Alsina, Ana Belén Custodio, Lourdes Fernández Franco, Javier Gallego Plazas, Carlos Gómez-Martín, Paula Richart, Fernando Rivera, Marta Martin-Richard

**Affiliations:** 1 Medical Oncology Department, Complexo Hospitalario Universitario de Ourense, Ramón Puga, 56, 32005 Ourense, Spain; 2 Medical Oncology Intercenter Unit, Regional and Virgen de la Victoria University Hospitals, IBIMA, Málaga, Spain; 3 Medical Oncology Department, Hospital Universitario de Navarra (HUN), Pamplona, Spain; 4 Vall d’Hebron Institute of Oncology (VHIO), Barcelona, Spain; 5 Medical Oncology Department, Hospital Universitario la Paz, CIBERONC CB16/12/00398, Madrid, Spain; 6 Medical Oncology Department, Hospital Virgen de la Salud, Toledo, Spain; 7 Medical Oncology Department, Hospital General Universitario de Elche, Elche, Alicante, Spain; 8 Gastrointestinal Cancer Unit, Medical Oncology Division, Doce de Octubre University Hospital, Madrid, Spain; 9 Medical Oncology Department, Hospital Universitari I Politècnic la Fe, Valencia, Spain; 10 Medical Oncology Department, Hospital Universitario Marqués de Valdecilla and Instituto de Investigación Marqués de Valdecilla (IDIVAL), Santander, Spain; 11 Medical Oncology Department, Institut Català d’Oncologia Hospital Duran i Reynals, CIBERONC, Barcelona, Spain

**Keywords:** Esophageal cancer, Diagnosis, Treatment

## Abstract

Esophageal cancer is an aggressive tumor, and is the sixth-leading cause of death from cancer. Incidence is rising in Spain, particularly among men. Two main pathological different subtypes have been described: squamous cell carcinoma and adenocarcinoma. Growing evidence of their epidemiology and molecular differences explains their different response to novel treatments, and they are therefore likely to be treated as two separate entities in the near future. The best results are obtained with a multidisciplinary therapeutic strategy, and the introduction of immunotherapy is a promising new approach that will improve prognosis. In these guidelines, we review the evidence for the different methods of diagnosis and therapeutic strategies that form the basis of our standard of care.

## Introduction, epidemiology, localization, histology and molecular biology

Esophageal cancer (EC) is a major public health problem. It is the seventh most common cancer worldwide, and the sixth-leading cause of death from cancer, with a 5-year survival rate of only 15–20%. In Spain, incidence is expected to increase from an estimated 2368 new cases in 2021 [1] to more than 2900 new cases in 2040 [2]; 70% of all cases occur in men. The survival rate in Spain varies depending on gender: rates increased by 35% in men and decreased by 9% in women between the 2002–2007 and 2008–2013 periods [1].

There are two main histological subtypes: squamous cell carcinoma (SCC) (84% of new cases) located predominantly in the upper and mid-esophagus, and adenocarcinoma (ADC) (15%), predominantly in the lower esophagus. Although the squamous subtype is the most prevalent, especially in Asia and Eastern Africa, its worldwide incidence has decreased in recent decades [3]. The incidence of adenocarcinoma, however, continues to rise sharply in developed countries, and now exceeds squamous cell rates in North America, Europe and Australia. These differences in incidence, trend, and geographical distribution reflect the particular etiology attributed to each subtype: alcohol and tobacco are mainly related to squamous cell carcinoma, meanwhile gastro-esophageal reflux and central obesity are important risk factors for Barrett’s esophagus (a precursor lesion) and adenocarcinoma.

Squamous cell carcinoma of the esophagus has molecular features that are similar to those of squamous cell carcinoma of the head and neck, while esophageal adenocarcinoma is more similar to gastric adenocarcinoma in terms of its chromosomally unstable subtype. In squamous cell carcinoma, alterations in cell cycle regulators with inactivation of CDKN2A and amplification of CCND1 are frequent [4]. Three molecular subtypes of esophageal squamous cell carcinoma (ESCC) have been described: ESCC1, with mutations of NRF2, associated with a poor prognosis and resistance to chemo-radiotherapy; ESCC2, in which NOTCH1 or ZNF750 are frequently mutated; and ESCC3, characterized by alterations that activate the PI3K pathway. Expression of programmed death-ligand 1 (PD-L1) is higher in squamous cell carcinoma than in adenocarcinoma [5]. In contrast, E-cadherin expression and upregulation of ARF6, FOX A and MAPK pathways are characteristic of adenocarcinoma, in which overexpression and amplification of ERBB2 are common features.

## Diagnosis and staging

Confirmatory diagnosis of esophageal cancer relies on endoscopic biopsy, and histology must be performed according to the WHO criteria.

Once the histological diagnosis is established, the clinical stage must be determined to establish the prognosis and define the therapeutic strategy.

To complete the study, the ECOG scale must be determined and a complete physical examination, with a comprehensive geriatric assessment in the case of elderly patients, must be performed. A nutritional assessment is also necessary, and patients must be given nutritional advice. It is essential to collect samples for blood analysis, including hemogram and renal panel, and imaging tests (CTScan of chest and abdomen) are also a requirement. In patients who are candidates for surgery or radical treatment, consider expanding the study with:

- Echoendoscopy [6] (II,A).
- PET-CT or PET may detect metastases not evidenced on CT in 10–20% of patients [7] (III,A).
- Bronchoscopy: if the tumor is located above or in the tracheal bifurcation (II A).
- In locally advanced tumors (T3/4) in the distal esophagus or esophago-gastric junction (EGJ) with adenocarcinoma histology, an exploratory laparoscopy and peritoneal cytology must be performed, given that 15% of patients present hidden peritoneal metastases (IV,B) [8].

The disease should be staged according to the 2017 UICC-AJCC classification (8th edition) (Table 1) and categorized according to histology (Table 2) [9].

**Table 1:** TNM staging for esophageal and esophagogastric junction (EGJ) cancer (AJCC/UICC 8th edition)

T- primary tumor	
Tx	Primary tumor cannot be assessed
T0	No evidence of primary tumor
Tis	Carcinoma in situ/high-grade dysplasia
T1	Tumor invades lamina propia, muscularis mucosae, or submucosa
T1a	Tumor invades lamina propia or muscularis mucosae
T1b	Tumor invades submucosa
T2	Tumor invades muscularis propia
T3	Tumor invades adventitia
T4	Tumor invades adjacent structures
T4a	Tumor invades pleura, pericardium, azygos vein, diaphragm, or peritoneum
T4b	Tumor invades other adjacent structures such us aorta, vertebral body, or trachea
N- regional lymph nodes	
Nx	Regional lymph nodes cannot be assessed
N0	No regional lymph node metastasis
N1	Metastasis in 1 to 2 regional lymph nodes
N2	Metastasis in 3 to 6 regional lymph nodes
N3	Metastasis in 7 or more regional lymph nodes
M- distant metastasis	
M0	No distant metastasis
M1	Distant metastasis
G- histology grade	
Gx	Unknown grade
G1	Well differentiated
G2	Moderately differentiated
G3	Poorly differentiated o undifferentiated

**Table 2:** Stage grouping according to histology

Squamous cell carcinoma						Adenocarcinoma				
Group	T	N	M	Grade	Location	Group	T	N	M	Grade
0	Tis	N0	M0	N/A	Any	0	Tis	N0	M0	Any
IA	T1a	N0	M0	1,X	Any	IA	T1a	N0	M0	1, X
IB	T1a	N0	M0	2–3	Any	IB	T1a	N0	M0	2
	T1b	N0	M0	Any	Any		T1b	N0	M0	1, 2
	T2	N0	M0	1	Any					
						IC	T1a,b	N0	M0	3
							T2	N0	M0	1,2
IIA	T2	N0	M0	2,3,X	Any	IIA	T2	N0	M0	3,X
	T3	N0	M0	Any	Lower					
	T3	N0	M0	1	Upper, middle					
IIB	T3	N0	M0	2,3	Upper, middle	IIB	T1	N1	M0	Any
	T3	N0	M0	Any	X		T3	N0	M0	Any
	T3	N0	M0	X	Any					
	T1	N1	M0	Any	Any					
IIIA	T1	N2	M0	Any	Any	IIIA	T1	N2	M0	Any
	T2	N1	M0	Any	Any		T2	N1	M0	Any
IIIB	T2	N2	M0	Any	Any	IIIB	T2	N2	M0	Any
	T3 N1,2 M0 Any	Any						T3 N1,2	M0	Any
	T4a	N0	M0	Any	Any		T4a	N0	M0	Any
IVA	T4a	N2	M0	Any	Any	IVA	T4a	N2	M0	Any
	T4b	Any N	M0	Any	Any		T4b	Any N	M0	Any
IVB	Any T	Any N	M1	Any	Any	IVB	Any T	Any N	M1	Any
(8)										

## Treatment

Initial treatment approaches for esophageal cancer and EGJ depend on several factors, and each case should be discussed by a multidisciplinary team (Table 3).

**Table 3:** Diagnosis and treatment

General	Details	Quality of evidence	Strength of recommendation
**Diagnosis and staging**			
Gastroscopy CT scan Endoscopic ultrasound (EUS + – fine needle aspiration (FNA) 18F-FDG positron emission tomography (PET) or PET-CT (preferred) Bronchoscopy Staging laparoscopy and peritoneal cytology	In tumors at or above the tracheal bifurcation In locally advanced (T3/T4) distal esophageal or esophagogastric junction (EGJ) adenocarcinomas	II	A
		III	B
		II	A
		IV	B
**Treatment**			
*Early stage*			
T1a N0 (< 2 cm, well or mod. differentiated)	Endoscopic resection	III	A
	Surgery	IV	A
T1b-2N0	Surgery	I	A
*Locally advanced disease (T3-4 N0; T1b-T4a, N +)*			
Cervical spine tumors	Cisplatin- Fu + RDT	II	A
T1–2 N +	Preop Paclitaxel-carbo + RDT and surgery	I	A
T3–4 aN0– 2	*SCC*		
	Preop (Paclitaxel -carbo) or (Cisplatin- FU) + RDT and surgery	I	A
	Definitive CT + RDT + – salvage surgery	II	B
	Neoadjuvant CT and surgery	II	B
	*ADC*		
	Preop (Taxol-carbo) or (Cisplatin-FU) + RDT and surgery	I	A
	Neoadjuvant CT and surgery (distal tumors)	I	A
	*Adjuvant treatment if no pRC*		
	Nivolumab	I	A
T4bN0–2	*Fit patients*		
	Definitive Cisplatin-FU + RDT	I	A
	*Unfit patients*		
	Oxaliplatin-Fu + RDT	II	B
	Paclitaxel–Carboplatin + RDT	III	B
*Metastatic carcinoma*	*1st-Line CT*		
	*SCC*		
	Platinum-Fluoropyrimidine	II	A
	CT-Pembrolizumab if CPS ≥ 10	I	A
	*ADC*		
	Platinum-Fluoropyrimidine	I	A
	CT-Nivolumab if CPS 5	I	A
	CT-Pembrolizumab if CPS ≥ 10	II	A
	CT and trastuzumab (Her 2 +)	II	A
	*2nd-line and beyond CT*		
	Doublet CT or monotherapy (irinotecan or taxane)	II	B
	Nivolumab, pembrolizumab, camrelizumab, tislelizumab	I	A
	Paclitaxel and Ramucirumab only ADC	II	A
	Trifluridine and tipiracil only ADC	II	A

Evidence and recommendations

*SCC* Scamous cell carcinoma, *ADC* Adenocarcinoma, *pRC* patologic complete response

### Early disease (cT1-T2 cN0M0)

Depending on their depth of infiltration, mucosal carcinomas (T1a) are subdivided into m1, m2 and m3, and submucosal carcinomas (T1b) are classified as sm1, sm2 and sm3. While lymph node metastases are very rare in mucosal carcinomas, in submucosal cancers, their incidence increases as infiltration reaches deeper layers [10].

Endoscopic resection (ER) may be performed in early-stage cancers, provided it is indicated: high-grade intraepithelial neoplasia or mucosal carcinoma (adenocarcinoma: m1–m3, squamous cell carcinoma: m1–m2), no lymph or vascular invasion (L0/V0 status), no ulcerations and well or moderately differentiated (G1/G2) (III, A). ER is also possible in superficial (< 500 µm) adenocarcinoma infiltration in submucosal carcinomas (sm1) measuring less than 20 mm that do not meet the aforementioned risk criteria, but outcomes are poorer than in mucosal carcinoma (IV, B). After curative ER, the surrounding Barret´s mucosa should be ablated to prevent metachronous lesions (III, A). Unlike adenocarcinoma, squamous cell carcinoma with deep mucosal (m3) and submucosal infiltration is an indication for surgery [11] (V, A). Although no randomized controlled trials have compared ER with surgery in early disease, retrospective series have shown that endoscopic procedures were an alternative that was associated with shorter hospital stays, fewer readmissions, and lower 90-day mortality, but survival data in these studies were inconsistent. A recent meta-analysis concluded that ER is safe in early-stage esophageal cancer, but esophagectomy may be associated with better long-term survival. ER, therefore, cannot completely replace radical surgery; however, outcomes are favorable in suitable indications when accurate clinical staging is carried out.

Surgery remains the gold standard for T1b-T2 tumors and for mucosal carcinomas with high risk factors after ER (extensive carcinoma in situ, high-grade, lymphovascular invasion or positive deep margins). Subtotal transthoracic esophagectomy (Ivor Lewis esophagectomy) with 2-field lymphadenectomy is the procedure of choice [12] (I, A). If the carcinoma is located in the upper thorax, total esophagectomy with cervical anastomosis (McKeown esophagectomy) may be needed. Minimally invasive procedures are becoming more widespread in clinical practice, and are expected to reduce postoperative morbidity.

The value of preoperative treatment in limited disease is unclear, since only small randomized trials have been performed to date. Several studies found that neoadjuvant therapy compared to surgery alone had no significant effect on survival or recurrence, despite significant downstaging and a higher rate of pathological complete response (pCR) [13, 14]. These results suggest that surgery should be recommended as the primary treatment (I,A). However, there is evidence that a subset of these patients are under-staged and would benefit from neoadjuvant therapy. At this point, an open discussion with the patient should guide the shared decision process.

### Locally advanced disease

#### Cervical esophageal cancer

Most cervical esophageal cancers cannot be treated with surgery, as this would involve mutilating resections, such as pharyngo-laryngo-esophagectomy. Therefore, definitive chemoradiation therapy with curative intent is the standard treatment modality [14] (II, A). Persistent or locally recurrent cancer can be treated with salvage surgery, which has a higher risk of morbidity but is the only option for relatively long-term survival [15] (IV, B).

#### Neoadjuvant chemotherapy treatment

Neoadjuvant chemotherapy (CT) is less clinically developed than chemoradiotherapy (CRT). Earlier studies give it priority over adjuvant therapy in squamous cell carcinoma (I A), and it could be recommended before surgery (I,B).

Neoadjuvant chemotherapy showed statistically significant better overall survival results than adjuvant chemotherapy in the JCOG9907 phase III clinical trial in squamous cell carcinoma of the esophagus (five-year overall survival rate 55% vs 43 %; HR 0.73; 95% CI 0.54–0.99) [16] (I, A).

Preoperative chemotherapy may improve survival compared to surgery alone. Although the INT 113 study, the largest carried out so far by the UK Medical Research Council with 802 patients (66% adenocarcinoma, 31% squamous cell carcinoma), did not show OS improvement with CT, it did show increased five-year overall survival with this strategy (14% in the surgery only group vs 19% in the preoperative chemotherapy group; HR 0.84, 95% CI 0.72–0.98), and no differences in postoperative complications or mortality [17] (I, B).

Neoadjuvant chemotherapy has not demonstrated inferiority in overall survival compared to preoperative chemoradiotherapy, although complete pathologic response was higher in the chemoradiotherapy group (23% of patients with adenocarcinoma, 42% with squamous cell carcinoma) in the NeoResl trial and in the Burmeister et al. phase II trial (only adenocarcinoma, including gastroesophageal junction; rate favoring chemoradiotherapy; no difference in OS in toxicity and surgical complications) [18] (II, B).

#### Neoadjuvant chemoradiotherapy

Preoperative CRT is the standard treatment in operable patients with locally advanced esophageal cancer, since a survival benefit as well as an increase in R0 resections from preoperative CRT over surgery alone has been confirmed in several randomized trials and at least 1 network meta-analysis (I, A) [19–21].

The optimal radiation dose for preoperative CRT regimens is not well defined, although a total dose of 41.4–50.4 Gy administered in daily 1.8 Gy fractions, 5 days per week gives reasonable results with acceptable toxicity (II, A).

##### Squamous cell carcinoma

Various meta-analyses and 2 well-designed phase III randomized trials support CRT as the standard of care for patients with locally advanced esophageal squamous cell carcinoma (I, A).

In the European CROSS trial, which included 363 patients with resectable esophageal or esophagogastric junction (EGJ) cancer (86 SCC, 273 adenocarcinoma), preoperative CRT using weekly paclitaxel 50 mg/m2 plus carboplatin (area under the curve of concentration × time [AUC] of 2) plus concurrent RT (41.4 Gy over 5 weeks) results in a significant increase in the R0 resection rate with a 29% of pCR. Median overall survival was significantly better with preoperative CRT (HR for death 0.657, 95% CI 0.495–0.87), and this benefit persisted after a 10-year follow-up period (38% vs. 25% in the surgery only arm, HR for death 0.70, 95% CI 0.55–0.89 [21].

Similarly, the Chinese NEOCRTEC5010 trial randomly assigned 451 patients with potentially resectable thoracic ESCC to neoadjuvant CRT, (vinorelbine 25 mg/m2 iv on days 1 and 8 and cisplatin 75 mg/m2 IV day 1, or 25 mg/m2 IV on days 1 to 4 every 3 weeks for 2 cycles, with a total concurrent radiation dose of 40.0 Gy administered in 20 fractions of 2.0 Gy on 5 days per week), or surgery alone. In the CRT arm, the pCR rate (43%) as well as the R0 resection rate was higher than in the surgery only approach, and neoadjuvant therapy was associated with better five-year overall and disease-free survival [22].

In the CROSS or NEOCRTEC5010 trials, postoperative morbidity or mortality did not differ between CRT and surgery only arms.

Several meta-analyses have explored trimodality therapy over surgery alone for esophageal cancer [19, 23], and have found the benefit in terms of survival to be similar across histologic subtypes (SCC, HR 0.80, 95% CI 0.68–0.93; adenocarcinomas, HR 0.75, 95% CI 0.59–0.95).

##### Adenocarcinoma

Preoperative CRT (41.4–50.5 Gy in fractions of 1.8–2.0 Gy/day combined with cisplatin/5-F, or carboplatin/paclitaxel) should be considered standard treatment in locally advanced adenocarcinoma of the esophagus (I,A). In some studies, peri-operative chemotherapy with a platinum/ fluoropyrimidine regimen for 2–3 cycles pre- and post-surgical resection of the primary tumor were included.

The benefits of CRT followed by surgery are based on results from meta-analyses and some large randomized trials [20, 23]. However, direct comparisons of chemotherapy versus CRT as neoadjuvant therapy are limited to small randomized trials [24], and there are no studies specifically comparing trimodality versus bimodality (CRT alone without surgery) in adenocarcinomas. Neoadjuvant CRT gives a higher rate of complete pathologic response as well as increased R0 rates without significant impact on survival when compared with neoadjuvant chemotherapy.

Despite complete tumor response to neoadjuvant treatment, the need for surgical resection is controversial, and data from retrospective analyses suggest inferior outcomes in patients with adenocarcinoma treated without surgery after CT or CRT. A survival benefit for surgery in SCC has also been suggested in large retrospective analyses (IV, B) and in non-responders [25].

#### Perioperative treatment

##### Adenocarcinomas

Based on the results of several randomized Phase III trials and metanalysis, preoperative CT could be considered an alternative to standard CRT in locally advanced adenocarcinoma of the esophagus (I,A) [26–28].

Perioperative chemotherapy compared to surgery alone demonstrated improvements in disease free survival (HR 0.65; 95 CI, 0.48–0.889), in progression-free survival (HR 0.66; 95% CI, 0.53–0.81) and in overall survival (HR 0.73; 95% CI 0.61–0.88) in two studies and a meta-analysis of patients with adenocarcinoma of the lower esophagus, gastroesophageal junction and stomach [26, 27] (I, A). Better overall survival results have been more recently achieved with the FLOT regimen, but only in resectable gastric or gastroesophageal adenocarcinoma (HR 0.77; 95% CI, 0.63–0.94) [28] (lack of evidence for esophageal cancer).

Perioperative chemotherapy demonstrated non-inferiority versus preoperative chemoradiotherapy in a recent phase III trial in patients with stage I-III esophageal and esophagogastric junction adenocarcinoma [29] (I, A).

##### Squamous cell carcinoma

There is less evidence for perioperative CT evidence in this setting (II,A)[30].

Perioperative chemotherapy has shown better relapse-free survival (HR 0.62; 95% CI, 0.49–0.73) and overall survival (HR 0.79; 95% CI, 0.59–0.95) without increasing toxicity in phase III clinical trial results compared with neoadjuvant chemotherapy in squamous cell carcinoma of the esophagus [30]. However, the lack of stratification by stages and surgical reports detracts from the conclusions reached (II,A).

#### Adjuvant treatment

Adjuvant nivolumab, if available, should be recommended as standard treatment in patients with residual disease in the esophagectomy specimen after neoadjuvant CRT (I,A).

The benefit of adjuvant nivolumab is derived from the Checkmate 577 trial [31], where 794 patients with residual esophageal cancer after neoadjuvant CRT were randomized, irrespective of histology or PD-L1 status. Median disease-free survival was twice as long with nivolumab vs placebo, and the benefits were seen across all patient subgroups. The potential benefit of this adjuvant treatment in patients undergoing perioperative chemotherapy instead of CRT remains unclear.

In patients with or without residual nodal disease following CRT and esophagectomy, the benefit of adjuvant chemotherapy in terms of survival is doubtful, and is based on two retrospective analyses (IV,C). The potential gain in survival should be balanced against poor tolerability of further cytotoxic chemotherapy.

The best approach in patients who have not received neoadjuvant therapy remains unclear. Evidence from uncontrolled trials and retrospective series suggests a potential benefit for adjuvant CRT, but there are no randomized trials proving benefit compared with surgery alone (V,B). Similarly, postoperative CRT (cisplatin/paclitaxel plus RT 50 Gy) improved overall survival and recurrence compared with RT alone in a retrospective review of patients with thoracic SCC who had undergone upfront esophagectomy.

Survival benefit from postoperative chemotherapy alone in patients not receiving preoperative chemotherapy or CRT is unclear. In the only randomized trial comparing surgery versus surgery followed by adjuvant chemotherapy (cisplatin/5 FU), improvement in disease-free survival did not correlate with an overall survival gain [32] (II,C).

### Locally advanced: inoperable, unresectable or surgery not planned

Definitive concomitant chemoradiotherapy with platinum and fluorouracil-based regimens without surgery have been shown to be superior to radiotherapy alone, and achieve long-term survival of around 30% (I,A) [33].

This is the only therapeutic option in tumors that, though localized, are in surgically inaccessible regions, such as the cervical esophagus, and in patients with comorbidities that rule out surgery even if their tumors were initially resectable.

The most commonly used regimen in this context is cisplatin combined with 5-FU (CF) [33]. Alternative approaches are FOLFOX (folinic acid, 5FU and oxaliplatin) [34] (II, B) or carboplatin and paclitaxel (III,B). The standard radiotherapy dose is 45–50.4 Gy, and higher doses of RT are no more effective and are more toxic [35, 36] (II,B).

#### Inoperable due to comorbidities

Patients that are inoperable due to their comorbidities, their general condition, or their age are not usually included in clinical trials, and therapeutic toxicity is usually more important, particularly in the more aggressive options. The best approach in these patients is probably a less toxic drug or even radiotherapy alone (V, B).

#### Unresectable

Special mention should be made of unresectable T4b tumors. When there is infiltration of large vessels (aorta, pulmonary arteries) or the trachea, the administration of radiotherapy is associated with a high risk of massive bleeding or tracheal esophageal fistulae, so its use is discouraged. In this situation, the administration of chemotherapy alone is the best option, and surgery can be evaluated when response is positive enough to reconsider resection. (V, B).

#### Surgery not planned

Another special mention refers to resecable and operable locally advanced disease. Definitive chemo-radiotherapy without planned surgery (with clinical response) can be considered. Surgery can be avoided in initially resectable and operable locally advancedsquamous cell carcinomas of the esophagus if a clinical response is achieved after chemo-radiotherapy, mainly in patients with comorbidities [37] (IIB). In adenocarcinomas of the esophagus, however, if the tumor is resectable and the patient is operable, surgery should always be considered as part of the therapeutic plan. (IV, B).

If definitive chemo-radiotherapy is attempted, surgical rescue should be evaluated in the case of only local persistence or recurrence [38] (III,B).

### Advanced/ metastatic disease

#### First-line chemotherapy

Patients with metastatic or inoperable locally advanced esophageal cancer have a poor prognosis, with a median overall survival (OS) of less than 1 year when treated only with chemotherapy. Despite differences in biology [4, 39], SCC and esophageal ADC have traditionally been treated with similar chemotherapy combinations, based on a platinum salt and a fluoropyrimidine [38, 40]. The recent introduction of immune checkpoint inhibitors in combination with chemotherapy has improved outcomes in these patients [41–44].

#### First-line treatment for esophageal squamous cell cancer

The standard first-line chemotherapy treatment is based on a platinum salt (cisplatin or oxaliplatin) and a fluoropyrimidine [5-fluorouracil or capecitabine] (II A). This recommendation is mainly based on extrapolation of clinical trials conducted in esophageal and gastric adenocarcinoma [40, 45, 46], and is routinely used.

In patients with programmed death ligand-1 (PD-L1) combined positive score (CPS) ≥ 10, the addition of pembrolizumab to a cisplatin and fluoropyrimidine regimen should be recommended (I,A).

The phase III KEYNOTE-590 trial included 749 esophageal and Siewert type 1 gastro-esophageal cancer patients, mostly ESCC (73%), randomized to receive pembrolizumab or placebo plus 5-fluorouracil and cisplatin [41]. Greatest OS improvement was observed in ESCC CPS ≥ 10 (13.9 vs 8.8 months; HR 0.57 [95% CI 0.43–0.75]; *p* < 0.0001). Although a significant benefit was demonstrated in ESCC regardless of the CPS status (OS of 12.6 vs 9.8 months; HR 0.72 [95% CI 0.60–0.88]; *p* = 0.0006), it disappeared in patients with CPS < 10. The CheckMate 648 trial (presented in abstract form) randomized 970 ESCC patients to receive nivolumab plus ipilimumab, nivolumab plus cisplatin and fluorouracil, or chemotherapy alone [42]. Patients treated with nivolumab and chemotherapy presented improved OS compared with those treated with chemotherapy alone; this effect was greater in patients with tumor cells PD-L1 (TPS) ≥ 1%. Patients treated with nivolumab plus ipilimumab also presented an OS benefit compared with chemotherapy alone; this gain was mostly restricted to those with TPS ≥ 1% tumors. Finally, the Chinese ESCORT-1 trial (presented in abstract form) also demonstrated an OS benefit in ESCC patients with the addition of camrelizumab to a first-line carboplatin and paclitaxel therapy [43].

Despite this evidence, immunotherapy is still awaiting financial approval from the health authorities in Spain.

#### First-line treatment for esophageal adenocarcinoma

The standard first-line chemotherapy treatment is based on a platinum salt (cisplatin or oxaliplatin) and a fluoropyrimidine (5-fluorouracil or capecitabine) (I,A). This recommendation is based on clinical trials conducted in gastric cancer and EAC patients. Oxaliplatin can replace cisplatin, and is generally less toxic. Capecitabine can replace 5-fluorouracil, and is a more comfortable option in the absence of dysphagia [40, 45, 46]. In patients with programmed death ligand-1 (PD-L1) combined positive score (CPS) ≥ 10, the addition of pembrolizumab to a cisplatin and fluoropyrimidine regimen should be recommended (II,A). In patients with programmed death ligand-1 (PD-L1) combined positive score (CPS) ≥ 5, the addition of nivolumab to an oxaliplatin and fluoropyrimidine regimen should be recommended (I,A).

Two phase III studies, one mentioned above, showed an improvement in OS with the addition of anti-PD1 to upfront chemotherapy**.** In the phase III KEYNOTE-590, 13% of patients (201) had CPS ≥ 10 adenocarcinoma, and the results demonstrated the benefit of adding pembrolizumab to chemotherapy (II,A). The phase III CheckMate 649 trial randomized 1581 non-HER2-positive gastric, gastro-esophageal junction (GEJ) and esophageal adenocarcinoma patients (13% with EAC and 8% with GEJ) to receive nivolumab plus ipilimumab, nivolumab plus oxaliplatin and fluorouracil (XELOX or FOLFOX), or chemotherapy alone. Patients with PD-L1 CPS ≥ 5 tumors benefited from the combination of nivolumab and chemotherapy compared with chemotherapy alone (OS of 14.4 vs. 11.1 months; HR 0.71 [98.4% CI 0.59–0.86]; *p* < 0.0001). This benefit was not demonstrated in patients with PD-L1 CPS < 5 tumors (unstratified HR for OS of 0.94 [0.78–1.13]).

Immunotherapy is still awaiting financial approval from the health authorities in Spain.

Considering the molecular similarities between gastric cancer and EAC and the results of the phase III TOGA trial (which included only HER2-positive gastric and gastro-esophageal junction cancer patients [47], the addition of trastuzumab to a cisplatin and fluoropyrimidine regimen could be considered in HER2-positive EAC patients. However, this approach is not authorized.

No other molecular targeted therapy has demonstrated sufficient efficacy in the first-line setting. This failure may be due in part to the molecular heterogeneity of these tumors and poorly designed clinical trials.

#### Second-line chemotherapy and new targeted drugs

Treatment selection depends on performance status, histologic type, symptom burden, first-line therapy and biomarker analysis. CT is the standard of care; however, this will probably change after the introduction of immunotherapy (nivolumab, pembrolizumab, camrelizumab). Single or combination CT not used in first line should be considered as part of second line therapy (II,B).

Patients with squamous cell carcinoma are routinely treated with second-line chemotherapy. Single-agent therapy containing agents not used in the first-line setting (weekly paclitaxel, docetaxel or irinotecan) are generally preferred over polychemotherapy [48]. Combination regimens (FOLFIRI, taxane-based therapy) are usually reserved for patients with a high symptom burden or a need for a higher response rate (II,B). Four phase III clinical trials in anti-PD-1 antibodies in eligible patients who have not received immunotherapy for advanced disease reported encouraging efficacy in patients with esophageal carcinoma who progressed on or were intolerant to 1 previous platinum-based CT (Table 4). In the KEYNOTE-181, the only study including a predominantly non-Asian population with both SCC and adenocarcinoma histologies, pembrolizumab showed a significantly longer duration of response and median overall survival than CT in patients with PD-L1 combined positive score (CPS) ≥ 10, but not in patients with SCC or in the entire intent-to-treat population. In a subset analysis of patients with CPS ≥ 10, the survival benefit was significant for SCC but not for adenocarcinoma [49]. The other 3 studies provided evidence of the superiority of nivolumab (ATTRACTION-3)[50], camrelizumab (ESCORT)[51] and tislelizumab (RATIONALE 302) [52] over CT in exclusively or predominantly Asian patients with SCC histology. Subgroup analyses in this 3 trials showed that clinical benefits of anti-PD-1 therapy were observed in all PD-L1 expression subgroups, but patients with higher PD-L1 expression appear to derive more benefit than those with low PD-L1 expression. Based on these data, pembrolizumab is approved by the FDA for advanced esophageal SCC patients with PD-L1 CPS ≥ 10 after failing 1 regimen [49] (I, A). Nivolumab [50] (and camrelizumab [51], or tislelizumab where available, should be considered as second-line therapy in SCC regardless of PD-L1 expression (I, A).However, these treatments are not yet approved by the Spanish healthcare authorities.

**Table 4:** Phase III clinical trials of second-line anti-PD1 therapies in esophageal cancer

Study	Study population	Treatment arms	ORR (%)	Median PFS (months)	Median OS (months)
KEYNOTE-181 Kojima T [49]	SCC (63.1%)/AC (36.9%) of esophagus/Siewert 1 GEJ 38.5% Asian, 61% ROW	Pembrolizumab (*n* = 314) vs Chemotherapy (*n* = 314)	PD-L1 CPS ≥ 10: 21.5% vs. 6.1% SCC: 16.7% vs. 7.4% All patients: 13.1% vs. 9.5%	PD-L1 CPS ≥ 10: 2.6 vs. 3 (HR, 0.73; 95% CI, 0.54–0.97) SCC: 2.2 vs. 3.1 (HR, 0.92; 95% CI, 0.75–1.13) All patients: 2.1 vs. 3.4 (HR, 1.11; 95% CI, 0.94–1.31)	PD-L1 CPS ≥ 10: 9.3 vs. 6.7 (HR, 0.69; 95% CI, 0.52–0.93; *p* = 0.0074) SCC: 8.2 vs. 7.1 (HR, 0.78; 95% CI, 0.63–0.96; *p* = 0.095) All patients: 7.1 vs. 7.1 (HR, 0.89; 95% CI, 0.75–1.05; *p* = 0.0560)
ATTRACTION-3 Kato K [50]	SCC of esophagus/GEJ 96% Asian patients	Nivolumab (*n* = 210) vs Chemotherapy (*n* = 209)	ORR: 19% vs. 22% Median DoR: 6.9 vs. 3.9 months	1.7 vs. 3.4 (HR, 1.08; 95% CI, 0.87–1.34)	10.9 vs. 8.4 (HR, 0.77; 95% CI, 0.62–0.96; *p* = 0.019)
ESCORT Huang J [51]	ESCC 100% Chinese patients	Camrelizumab (*n* = 228) vs Chemotherapy (*n* = 220)	ORR: 20.2% vs. 6.4% Median DoR: 7.4 vs. 3.4 months (HR, 0.34; 95% CI, 0.14–0.92; *p* = 0.017)	1.9 vs. 1.9 (HR, 0.69; 95% CI, 0.56–0.86; *p* = 0.00063)	8.3 vs. 6.2 (HR, 0.71; 95% CI, 0.57–0.87; *p* = 0.0010)
RATIONAL 302 Shen L [52]	ESCC 79% Asian patients, 21% Europe/North American patients	Tislelizumab (*n* = 256) vs Chemotherapy (*n* = 256)	ORR: 20.3% vs. 9.8% Median DoR: 7.1 vs. 4 months (HR, 0.42; 95% CI, 0.23–0.75)	Not reported	All patients: 8.6 vs. 6.3 (HR, 0.70; 95% CI, 0.57–0.85; *p* = 0.0001) PD-L1 CPS ≥ 10: 10.3 vs. 6.8 (HR, 0.54; 95% CI, 0.36–0.79; *p* = 0.0006)

*AC* adenocarcinoma, *CI* confidence interval, *CPS* combined positive score, *DoR* duration of response, *ESCC* esophageal squamous cell carcinoma, *GEJ* gastroesophageal junction, *HR* hazard ratio, *ORR* overall response rate, *OS* overall survival, *PFS* progression-free survival, *ROW* rest of the world, *SCC* squamous cell carcinoma

Despite scarce evidence, patients with adenocarcinoma of either the distal esophagus or gastroesophageal junction (GEJ) are usually managed according to the recommendations for gastric cancer, as described in previous guidelines [53]. For most HER2-negative patients, the RAINBOW study recommended the combination of paclitaxel and ramucirumab [54] (II, A). Trifluridine/tipiracil appears to be a reasonable third-line option, when available, based on the survival benefit shown in the TAGS study [55] (II,A).

In the case of HER2-overexpressing tumors with confirmed persistency of HER2 positivity after progression on a trastuzumab-containing regimen, trastuzumab deruxtecan can be considered the most promising second-line option on the basis of significant improvements in response and survival compared with standard CT, as shown in the DESTINY-Gastric01 and DESTINY-Gastric02 phase II clinical trials. The results of the phase III DESTINY-Gastric04 study are still pending. Finally, pembrolizumab is becoming an increasingly accepted approach for defective mismatch repair (dMMR) tumors and for those with high tumor mutational burden (≥ 10 mutations per megabase) [56] after progression on standard therapies. This recommendation is based on robust, lasting tumor response reported in multicohort, phase II studies. However, pembrolizumab has not yet been approved for this indication in Spain.

MSI seems to be a clear predictive factor of response to immunotherapy in EAC (based on phase II trials and post hoc analysis of the population analyzed in pivotal phase III clinical trials) [57] (III, A).
